# Latitudinal Body Size Clines in the Butterfly *Polyommatus icarus* are Shaped by Gene-Environment Interactions

**DOI:** 10.1673/031.008.4701

**Published:** 2008-06-05

**Authors:** Georg H. Nygren, Anders Bergström, Sören Nylin

**Affiliations:** Department of Zoology, Stockholm University, 106 91 Stockholm, Sweden

**Keywords:** plasticity, life history, countergradient variation, Bergmann's rule, Lycaenidae

## Abstract

The study of latitudinal body size clines can illuminate processes of local adaptation, but there is a need for an increased understanding of the relative roles of genetic variation, environmental effectstions or this reason, we combined an investigation of a museum collection of the common blue butterfly *Polyommatus icarus* (Rottemburg) (Lycaenidae: Polyommatini) from Sweden with a common-garden experiment in the laboratory, using strains reared from individuals collected from three different latitudes. Sizes of the field-collected butterflies tended to smoothly decrease northwards in a latitudinal cline, but suddenly increase at the latitude where the life cycle changes from two to one generations per year, hence allowing more time for this single generation. Further north, the size of the field-collected butterflies again decreased with latitude (with the exception of the northernmost collection sites). This is in accordance with the “converse Bergmann” pattern and with the “saw-tooth model” suggesting that insect size is shaped by season length and number of generations along latitudinal transects. In contrast, under laboratory conditions with a constant long day-length there was a different pattern, with the butterflies pupating at a higher mass when individuals originated from southern populations under time stress to achieve a second generation. This is indirect evidence for field patterns being shaped by end-of-season cues cutting development short, and also suggests counter-gradient variation, as butterflies from the time-stressed populations over-compensated for decreasing larval development time by increasing their growth rates, thus obtaining higher mass. Hence, we found support for both adaptive phenotypic plasticity and local genetic adaptation, with gene-environment interactions explaining the observed field patterns.

## Introduction

The study of latitudinal life history trends within species has a strong potential to illuminate processes of local adaptation, which in turn can provide information on how populations may respond to factors such as climate change. It might be expected that geographical gradients in climate, and in length of the favourable season, should translate to biological gradients (for instance clines in animal life history traits) in a fairly predictable manner. To the extent that this is not the case would be evidence of our imperfect understanding of the processes behind local adaptation. As long as we cannot predict trends in the characteristics of organisms along such relatively simple gradients, we have little hope of predicting or even understanding biological variation in cases where the underlying environmental causes are less evident. That said, the study of latitudinal and altitudinal variation has proven to be a much more complex and challenging subject than perhaps was originally thought when various “rules of thumb” regarding geographical variation was first proposed.

One of the best known intra-specific geographical patterns in animals is “Bergmann's Rule”, depicting a size trend with larger size at higher latitudes as is found especially in the endothermic mammals and birds (Ashton et al. 2000). The underlying selection is in this case generally assumed to be related to thermoregulation; the allometric relationship between body mass and surface area selects for bigger animals in cold areas. It has been suggested that larger size at higher latitudes in ectotherms may be due to non-adaptive responses to low temperatures (Van Voorhies 1996), but adaptive explanations can also be found to explain increased size with latitude, other than thermoregulation. For instance, Boyce (1979) argued that an increased degree of seasonality with latitude can select for increased body size.

In insects and other arthropods the opposite size trend, i.e. decreasing size with increasing latitude (or altitude), has often been reported; this is the so-called “converse Bergmann's rule” (Mousseau 1997), that has been well documented (Mousseau and Roff 1989; Nylin and Svärd 1991; Blanckenhorn and Fairbairn 1995; Telfer and Hassall 1999; Johansson 2003). Still, some reports described an increase in size with increasing latitude also occurring in arthropods (Chown and Gaston 1999; Blanckenhorn and Demont 2004). There is clearly a need to explore the causes of this variation (Chown and Gaston 1999; Johansson 2003; Blanckenhorn and Demont 2004).

A possible explanation for the occurrences of variable patterns of body size with latitude in insects was suggested by Chown and Gaston (1999). They emphasised that species with short life spans, inhabiting ephemeral habitats, could be expected to increase their mass at higher latitudes. For such species the length of the favourable season does not set the limits for development time, but instead e.g. the availability of food resources. Larger size in colder areas could thus follow. In contrast, insects with a generation length more similar in magnitude to the length of the season can instead be expected to be constrained by season length and decrease their mass at high latitudes and altitudes. These predictions were later supported in a review of Blanckenhorn and Demont (2004).

The most elaborate theory of geographical size patterns in insects is that developed by Roff (1980, 1983), and it is compatible with the hypotheses of Chown and Gaston (1999) and Blanckenhorn and Demont (2004). Roff suggested that populations with many generations per year may often show an r-maximizing strategy (as is often implicitly assumed in insect optimality models), but that species or populations with few generations are better described as maximizing Ry, the yearly rate of increase. The reason is that insects generally can spend the winter only in a specific developmental stage, so that whole generations have to fit into the favourable season. Hence, Roff predicted that when the available time for growth decreases, the insect will be selected to mature earlier at the cost of a reduced adult size that could also reduce fecundity. This scenario results in a detectable pattern in development time and size along an environmental gradient; with “either a monotonic increase with ‘season length’ … or a ‘saw-tooth’ pattern (Roff 1983).

**Figure 1.**
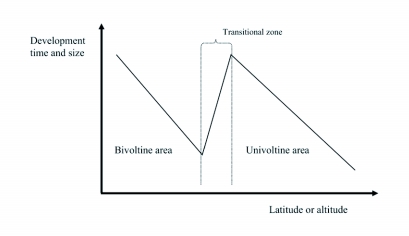
Theoretical “saw-tooth” pattern. After Roff (1980, 1983) and Nylin and Svärd (1991).

The saw-tooth pattern (Figure 1) is predicted to occur when a species shifts strategy from, for example, bivoltine (two generations per year) to univoltine (a single generation) within a study area. Insects in the bivoltine population near the transition zone to the univoltine zone live under time stressed conditions since their favourable season has effectively been cut in two parts, with the theoretical result of short development time and small individuals as elements of local life history adaptation. Conversely, the univoltine populations near the transition zone to the bivoltine area will have surplus time, and hence can develop for a longer time period to reach a bigger size. Size patterns wholly or partly consistent with such saw-tooth patterns have been described in some insect species (Masaki 1978; Mousseau and Roff 1989; Nylin and Svärd 1991; Mousseau 2000; Johansson 2003). See also Petersen (1947; e.g. pp. 438–439) for what is probably the first mention of an idea fully in line with Roff's hypothesis, introduced to explain the complex latitudinal size patterns in the butterfly *Pieris napi* in Sweden.

Observed field patterns can be due to complex interactions between Bergmann and converse Bergmann trends (Blanckenhorn and Demont 2004), further complicated by shifts in voltinism. In addition, there may be counter-gradient variation in growth rates (Conover and Schultz 1995) which has the potential to erase field trends in size even though, or because, genetic differences are evident in common-garden experiments. Over- or undercompensating counter-gradient variation is a further possibility and can result in field size trends that are difficult to predict (Blanckenhorn and Demont 2004).

Butterflies are known to frequently adjust their developmental time and growth rate according to photoperiod and temperature, and mass/size may also be a plastic trait (e.g. Wiklund et al. 1991; Leimar 1996; Gotthard 1998; Fischer and Fiedler 2000). Plastic growth rates have the power to uncouple development time from final size (Wiklund et al. 1991; Nylin 1992; Abrams et al. 1996; Davidowitz and Nijhout 2004) creating a three-dimensional time-rate-size relationship rather than the positive correlation between time and size explicitly assumed by Roff (1980, 1983). It is thus of special interest to determine whether insects with plastic growth rates, such as butterflies, still follow the patterns predicted by Roff and, if so, how such patterns are shaped by genetic or plastic variation among sites, or by gene-environment interactions. To this end, and as a test of the prediction that latitudinal trends in insects with generation lengths comparable to season length should show size trends explained by season length (Chown and Gaston 1999), we examined collections of field-caught individuals of the common blue butterfly, *Polyommatus icarus* (Rottemburg) (Lycaenidae: Polyommatini), and then reared strain from three different latitudes in a common environment. We found that in the field there is a pattern consistent with the saw-tooth model, but in the laboratory (with constant daylength and temperature) we found a different pattern, presumably resulting from counter-gradient genetic variation. This suggests that field size patterns in this butterfly are shaped by an interaction between genetic life history differences and environmental end-of-season cues.

## Materials and methods

### Studied species

The larvae of *P. icarus* feed on small herbs in Fabaceae, and the species spends the winter in a late larval instar. It is commonly found in grasslands and other open areas over the whole of the European continent, except for some islands on the northern tip of Scandinavia at latitudes > 70° N (Dal 1978). Lycaenids are small butterflies generally considered to be relatively sedentary (Hughes 2000). Therefore we assumed that *P. icarus* would have the opportunity to be locally adapted and show local genetic life history adaptations to climatic gradients, as was earlier found in some other lycaenids (Nylin and Svärd 1991).

**Figure 2.**
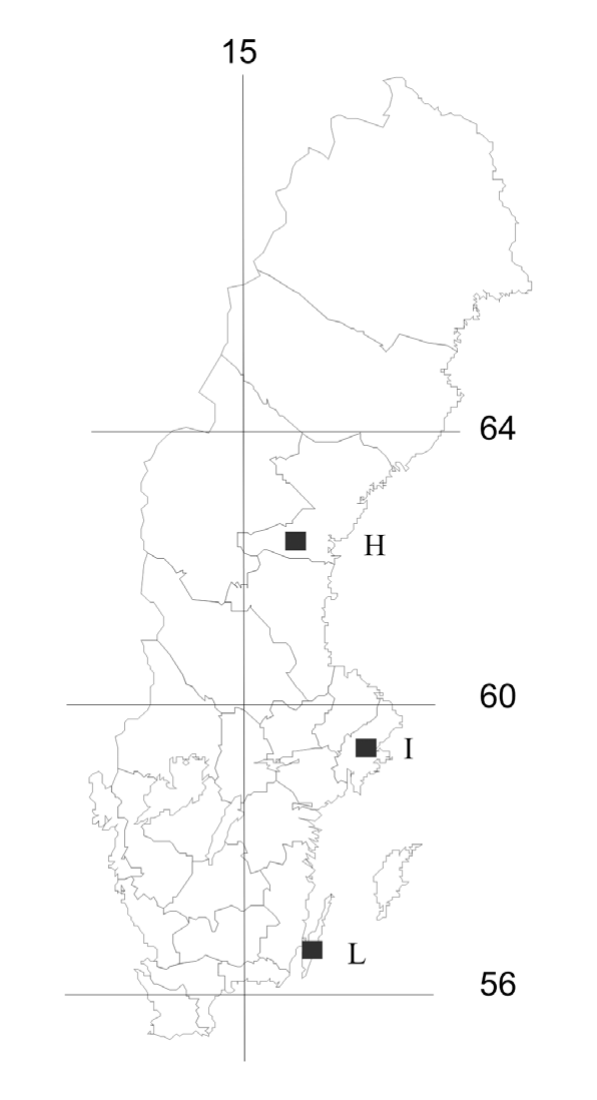
Map of Sweden depicting the source populations for the common-garden experiment: L = low latitude (Öland), I = intermediate latitude (Stockholm), H = high latitude (Borgsjö). One longitude (degrees East, vertical line) and three latitudes (degrees North, horizontal lines) given.

*P. icarus* is known to have two generations per year in Southern Sweden, e.g. on the Baltic island of Öland (locality L in Figure 2; Professor O. Leimar, Stockholm University, personal communication). We initially assumed that the species is univoltine in Northern Sweden, with a transition zone in Central Sweden, but the details regarding voltinism were revealed in a study of the museum collections of *P. icarus* (see below). *P. icarus* has been shown to respond to photoperiodic cues by plastically adjusting its size and development time according to perceived date in the season (Leimar 1996).

### Museum collection

The Swedish Natural History Museum has a collection of some 500 individuals of *P. icarus* collected from different latitudes in Sweden. The collection contains butterflies sampled between the years 1880 to 2001. Latitude and date of collection were determined from labels. Wing lengths (base to tip) of all individuals were measured under a standard laboratory microscope (6x). The average of right and left wings was used.

For purposes of illustrations and statistical analyses of size patterns, latitudes for collection sites were rounded off to nearest integer degree, whereas for purposes of studying the shift in generation number they were rounded off to nearest half degree. Rounding was done to increase sample sizes in latitudinal categories. We excluded the extreme latitudes of 55° and 69° due to very low sample sizes.

### Common-garden experiment

Females were collected in the wild from three populations for rearing of their offspring in the laboratory (Figure 2). Sixteen females were collected from the low-latitude population (two sites on Öland, 56.5° N), four from the intermediate population (Stockholm, 59.5° N) and two from the high-latitude population (Borgsjö, 62,5° N). In Britain *P. icarus* shows a shift from a predominantly bivoltine to a univoltine phenology approximately at latitude 54°, but with variation among years (Asher et al. 2001). In Sweden the transition zone seems to be even more ambiguous and individuals following a bivoltine pathway can regularly be found at higher latitudes than in Britain (Professor O. Leimar, personal communication, and see Results from the museum collection). However, the butterfly is clearly normally univoltine at the high-latitude site and generally bivoltine at the low-latitude site. In the intermediate area a second generation is not uncommon but neither is it a dominant strategy.

Length of the favourable season follows a strong and clear north-south pattern within Sweden (Nylin and Svärd 1991) due to a combination of factors: the effect of latitude itself combines with generally higher altitudes and more inland climates in the north. The low-latitude and the intermediate sites are situated close to the Baltic Sea. The high latitude sampling area is more continental, about 80 km inland and for instance has a visibly different flora compared to the coastal regions at the same latitude (Nygren, personal observation). This implies that despite the uniform latitudinal distances between the tree sampling areas, the high latitude sample is actually climatically more distant to the intermediate area than suggested by the geographical distance alone. The location of the low-latitude sites on an island should even further enhance the climatic distances between sites, in terms of day-degrees available in the favourable season.

To control for phenological changes in host plant quality, collected butterflies from all three sites were reared simultaneously. Wild-caught females and later eggs from low latitudes were stored for a short time period in a cold room to delay larval hatching and achieve synchronization. The experiment was initiated over a ten-day period in the second half of July. Newly hatched larvae were individually placed in plastic jars containing fresh bird's foot trefoil, *Lotus corniculatus* L. (Fabales: Fabaceae) and reared in a climate chamber at a photoperiod of 22: 2 LD at 22°C (low latitude n = 320, intermediate n = 52 and high n = 34). The plants were checked daily and changed when signs of wilting were found. Larvae of different latitudinal origin were mixed in the climate room to prevent position effects, and also moved in the room at least every second day.

Date of pupation was noted, and two days after pupation the pupal mass was measured and individuals were sexed. Wing length of adults was not taken for the laboratory-reared individuals, but pupal mass typically correlates well with adult weight and adult wing length among individuals within sexes in butterflies (Nylin 1992; Kemp 2000; Fric and Konvicka 2002) and moreover only differences among categories that were large enough to make the body measure chosen more or less irrelevant were used.

Growth rate was not measured independently of pupal mass, but for illustrative purposes a measure of growth rate was calculated as the natural logarithm of pupal mass divided by larval development time (in days).

All statistical analyses were performed with STATISTICA 1999 Edition, Kernel release 5.5.

## Results

### Museum collection

Above latitude 60° N, few *P. icarus* butterflies from a potential second generation, i.e. with a late collection date, could be found in the museum collection. At these latitudes, collecting dates were distributed approximately normally with a peak at mid-July. At latitudes below 60° N, the distributions of collecting dates had two peaks (late June and early August). Consequently, the butterfly appears to have shifted from a univoltine to a bivoltine life cycle at this latitude in Sweden. However, below latitude 60° the butterfly probably is only partly bivoltine as not all individuals develop directly, and this generation may not occur in all years at all latitudes (the collection was not large enough to permit further analysis of phenological details). It is unclear where an obligate bivoltine lifestyle takes over, if ever within Sweden, but below lat. 57.5° the second peak was consistently large, and hence bivoltinism appears to be the dominant strategy. These results confirmed our initial assumptions regarding where the bivoltine and univoltine populations would be found in the field.

**Figure 3.**
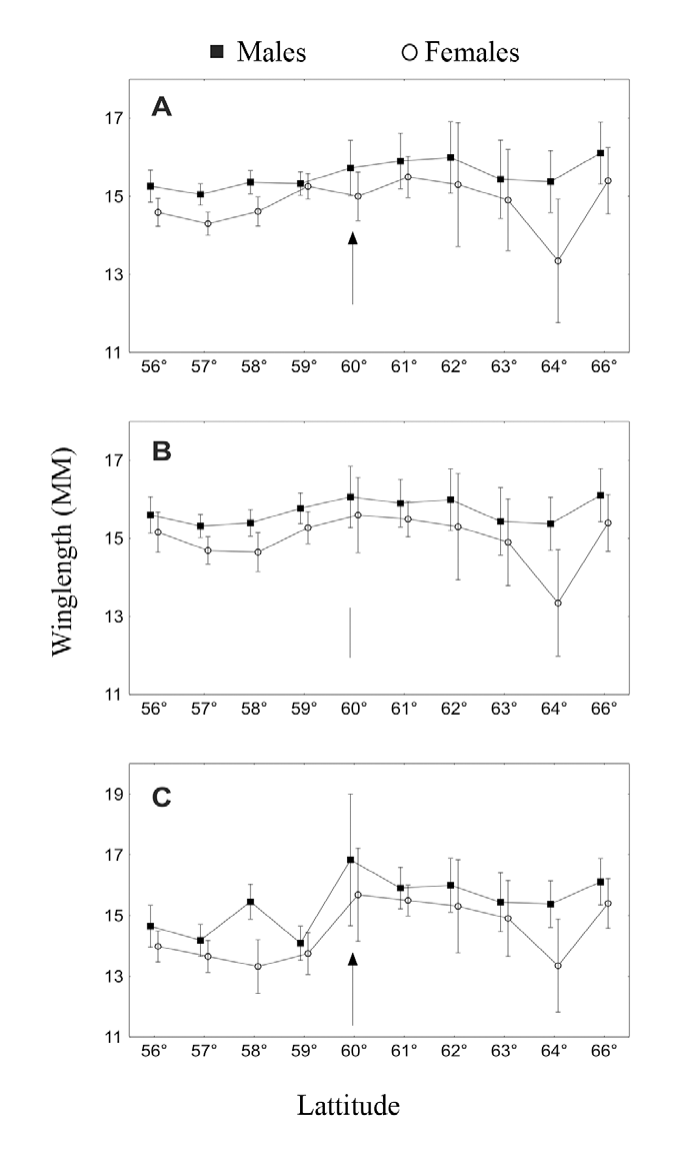
Wing length of *Polyommatus icarus* collected at different latitudes in Sweden. Arrows show the approximate shift from univoltine to bivoltine phenology, a) Average for both generations in the bivoltine area, b) only the over-wintering (first) generation in all areas, c) only the directly developing (second) generation in the bivoltine area. Bars show standard errors.

In the “bivoltine area”, below latitude 60° N, the butterflies were classified as belonging to either the first or the second generation. There were some individuals collected between the two peaks that may belong to either of the tails of the two overlapping generations or in some cases represent a single generation in a cold year. These butterflies were excluded from relevant analyses. Since the generations differed in size in bivoltine areas, we show results of depicting latitudinal patterns with these more southern areas represented by either all individuals (Figure 3a), only the first generation from over-wintering larvae (Figure 3b) or only the second, directly developing, generation (Figure 3c). In the field collection female size was consistently smaller (Figure 3a—c; GLM ANOVA, n = 522, p<0.001); for this reason the sexes are shown separately.

When all individuals from both generations were included there was no clear size pattern according to latitude (Figure 3a). When only insects with a history of hibernation were included there was a pattern faintly reminiscent of the predicted saw-tooth pattern (Figure 1), albeit with more smooth peaks and troughs (Figure 3b), and there was a significant effect of latitude on size (GLM ANOVA, n = 275 individuals, 10 latitudes, p<0.001; sex included as a factor). The butterflies were fairly large in the southernmost areas of Sweden, with size decreasing to the north. As predicted from the saw-tooth model, sizes increased again to a local maximum at around latitude 60° N, where the predominantly univoltine area begins, followed by a new decrease further north. Finally, size again increased at the very highest latitudes (Figures 3a—c). When only the second generation was chosen to represent the bivoltine areas (Figure 3c) the pattern was similar, but the maximum at latitude 60° N was even more evident. This is because wing length was always smaller for the directly developing generation than for the over-wintering generation in the bivoltine area below 60° N (compare Figures 3b—c; GLM ANOVA, n = 303 individuals, 4 latitudes, p<0.001; sex included as a factor).

### Common-garden experiment

Development time did not differ between butterflies reared from individuals collected from the two low latitude sampling areas, i.e. those on the island of Öland (t-test, p = 0.84). Pupal mass did differ significantly (t-test, p<0.05), but variation among latitudes was much larger. For simplicity of analysis and illustration, these two southern sites were therefore pooled in all statistical analyses and illustrations.

**Figure 4.**
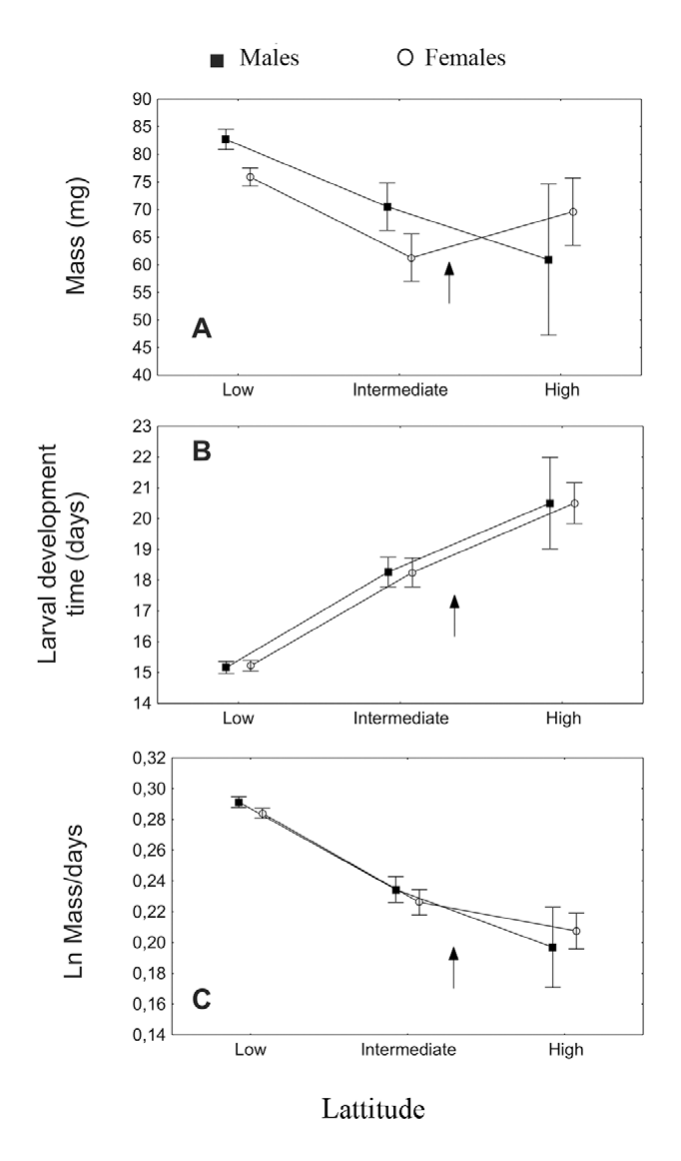
Results of the common-garden experiment with *Polyommatus icarus* originating from three different latitudes: Öland (Low=56.5° N), Stockholm (Intermediate=59.5° N) and Borgsjö (High=62.5° N). Arrows show approximate shift to a univoltine phenology, a) Mass (pupal weight), b) Larval development time, c) Growth rate calculated from the above. Bars show standard errors.

Female butterflies reared from individuals collected from southern and intermediate areas had lower pupal mass than males, but this was not true for the strain from the high latitudinal areas (Figure 4a); this interaction was however not statistically significant (GLM ANOVA, two-way interaction between sex and latitude as categorical predictors, n = 304, p=0.095). When the interaction term was removed, the effect of sex was significant (p<0.001). If latitude was instead treated as a continuous predictor, the interaction was significant (p<0.05), as well as the effects of sex (p<0.001) and latitude (p<0.001). The heaviest pupae were produced in the southern strain and the lightest in the northern strain (Figure 4a).

Interestingly, the latitudinal variation in mass did not correspond to variation in larval development time in the simple way assumed by Roff's theory on latitudinal patterns. Time spent in the larval stage differed significantly according to the latitudinal origin of strain (Figure 4b; GLM ANOVA, p<0.001). However, larval development time was in fact shortest in the southern strain and longest in the northern, with the intermediate strain showing intermediate values (Figure 4b). There was no significant effect of sex on larval development time (p = 0.96). Pupal development time differed significantly according to latitudinal origin (GLM ANOVA, p<0.05) but showing a pattern opposite to that for larval time (not shown). However, variation in pupal time was much smaller, so that the pattern for total development time was similar to that for larval time (effect of latitude p<0.001, not shown). The pattern of shorter development time yet larger mass in the southern areas corresponds to faster growth in southern strain, as illustrated in Figure 4c (GLM ANOVA, effect of latitude p<0.001). The interaction between sex and latitude can also be seen for growth rate, but it is not significant (p = 0.46). Effect of sex was not significant with the interaction included in the model (p = 0.76), but when the interaction was removed a significant difference could be seen, with males growing faster than females (p<0.01). If latitude was instead treated as a continuous predictor, the interaction was significant (p<0.05), as well as the effects of sex (p<0.01) and latitude (p<0.001).

There was a significant effect of family (p<0.001 in all cases) in the analyses described above, if family was added as a random factor nested in latitude, strongly suggesting a genetic component behind the observed variation. However, other results did not change; this is an indication that the geographical patterns may be robust even though there was a low number of families from two of the populations.

In the F1 generation no correlation between development time and mass among individuals could be found in the low (n = 253, p = 0.9) and intermediate (n = 41, p = 0.17) latitudinal sample, but in the high latitude strain they were positively correlated (n = 15, r^2^ = 0.36, p<0.05).

## Discussion

The intention was to test if the saw-tooth pattern in size according to latitude and generation number, predicted by Roff(1980, 1983), is present in the common blue butterfly *P. icarus* despite a lack of clear trade-offs between time and size in laboratory studies of this species (Leimar 1996). This is of particular interest in view of theory on three-dimensional time-rate-size relationships when growth rates are adaptively variable (Abrams et al. 1996; Nylin and Gotthard 1998). Acommon-garden experiment was also performed to obtain information on the genetic and plastic background of the phenotypic field pattern.

### Museum collection

The geographical position of the shift from a univoltine to a bivoltine life cycle by *P. icarus* in Sweden is somewhat ambiguous (see also Asher et al. 2001 regarding a similar situation for populations in Britain). This can, at least partly, be explained by gene flow, preventing exact local adaptation. Climatic variation among years is another likely cause; early instar larvae will respond in a plastic manner to cues signalling how much time is left in the season, with different outcomes in different years. In warm years this will result in two generations and in cold years in a single generation, so that the “transition zone” (as seen in the combined museum material) will extend over large geographical areas. This is not simply an artefact of pooling years, but in a real sense represents the temporally fluctuating environment to which local populations must adapt. In other words, most genetic differences between areas must reflect the probability of a second generation in a given area, rather than a fixed life cycle pattern.

The size of the *P. icarus* decreased towards higher latitudes with shorter seasons in the bivoltine area, but then increased when they shifted to a univoltine lifestyle, according to predictions. Interestingly, this pattern was not evident when the generations were pooled in the bivoltine area (as is the usual procedure when phenology is not clear, e.g. Nylin and Svärd 1991), because the generations differed in size (see also Fric et al. 2006). This size difference in itself strongly suggests that adult size is, in part, determined by plasticity, since the major developmental pathway - diapause or direct development - followed by an individual is an outcome of high-level plasticity according to seasonal cues such as photoperiod (Nylin and Gotthard 1998).

Observations of this kind also suggest that size trends in insects may have been underestimated in the scientific literature because of the difficulties introduced by generation differences, and furthermore introduce a problem regarding which pattern should be used to test Roff's hypotheses (1980, 1983). The pattern seen with only the first generation of adults (which has experienced larval hibernation) representing the bivoltine area (< 60° in Figure 3b) could be argued to be the most correct test. This is because the univoltine individuals further north (≥ 60°) always over-winter and they are thus more comparable to the first generation of adults in the south. Hibernation is associated with special demands on an insect and could incur metabolic costs visible as reductions in adult size or, conversely, larger size if winter survival is positively size-dependent (Kemp and Jones 2001).

On the other hand, it is the last generation of the favourable season that has the most direct information on how much time remains before winter in the bivoltine areas, and if size is partly plastically determined this generation may show the clearest size responses as a result of end-of-season cues from the environment (Roff 1983). Plasticity theory (Leimar et al. 2006) suggests that in such ambiguous cases, information from the environment should affect what phenotype will result from an individual's genotype. In a sense, the genotype of an individual organism provides it with information on what was the fittest life cycle in past environments, but only in a probabilistic way, and in some situations the current environment provides more precise information that should be exploited if possible.

Which generation should for these purposes be considered the “last of the year”, in the case of a bivoltine insect with late larval winter diapause such as *P. icarus* in Southern Sweden? This is not entirely clear either, but we suggest it is the second adult peak (Figure 3c). The first peak consists of individuals that spent the very last part of the previous summer as half-grown larvae and thus had a growth period at a later date than is ever the case for the second peak. However, the second peak consists of individuals that pupated later in the summer. Since growth of insects typically follows exponential or power functions, large size differences can occur due to small differences in the number of days spent in the last larval stage (Nylin 1994). This suggests that plastic size variation should mostly accumulate just before pupation, by the “decision” to continue growing or not as suggested by Leimar (1996) for *P. icarus*. The same conclusion can be reached based on the physiological model of body size variation in Lepidoptera presented by Davidowitz and Nijhout (2004), as they suggest that body size variation is the product of growth rate variation, coupled with variation in critical mass for pupation, and in the interval to cessation of growth, once the critical mass has been reached. The fact that the second generation is consistently smaller in size supports this interpretation, since it may suggest that this generation has to “pay the cost” of a bivoltine pathway by pupating early to ensure that enough time in the season remains for its offspring to reach the hibernating stage.

An unexpected pattern was found within the univoltine area. As predicted, sizes decreased going north within each of the areas of voltinism. However, further north in the univoltine area, *P. icarus* again increased its size, corresponding to field observations (K. Fiedler, University of Vienna, personal communication). If this pattern is to be explained by the saw-tooth theory, it would suggest that above latitude 65° *P. icarus* has adopted a two-year life-cycle (Johansson 2003). However, a clear possibility remains that environmental factors and biological responses other than those included in this theory come into play at such extreme latitudes.

### Common-garden experiment

Regarding the laboratory experiments, note that the low number of individuals used to create the strains for the intermediate and high latitude populations means that we cannot claim to have a good picture of the genetic make-up of these populations; more extensive sampling will be needed. Nevertheless, we believe that the differences between geographical localities may prove robust, for two reasons. First, there was little difference between two sites at the more extensively sampled low latitude locality. Second, adding family as a factor to statistical investigations did not change the overall results.

The highly significant effects of latitudinal origin of strain demonstrate that there is a strong genetic component to the observed life history variation within Sweden, not only in size/mass but also in development time and growth rate. The potential for local adaptation is also suggested by the apparent presence of genetic variation for these life history traits, visible as significant variation among families. The presence of genetic latitudinal patterns in a common garden environment lead us to propose that the life history observed in a given strain reflects adaptation to the probability that the source population (averaged over years) will have enough time to complete two generations. This probability may well increase to lower latitudes in a near-linear fashion over the area covered by the three populations, and we propose that the decreasing development times and increased growth rates in the lower latitudes (Figure 4b—c) reflect this higher probability of a second generation. At the high latitude site a second generation is very rare or non-existent, hence these butterflies probably always have more than enough time to complete the single generation per year (at even higher latitudes the situation might be different, see below). Accordingly, it is likely that at this site the butterflies have been selected for slow growth, since fast growth could be costly (Gotthard et al. 1994, Gotthard 2000). Conversely, at the site from the lowest latitude it is likely that *P. icarus* has been selected for fast growth, because there is generally enough time to complete two generations, but only barely.

The strain developed from individuals collected at the intermediate latitudes, were situated in a zone where development is not predictably uni- or bivoltine, and showed intermediate growth rates. This may be a result of selection from an intermediate probability of a second generation; however, gene flow from areas with more predictable life cycles probably also contributes. The most adaptive life history in this situation could be very fast growth under direct development (not the observed intermediate rates, because they have even less time than the southern population to complete two generations) and very slow growth under development to diapause. Although differences between developmental pathways in this general direction are commonly found in butterflies and other insects (Nylin and Gotthard 1998) gene flow and other constraints on reaching the optimal reaction norm (such as genetic correlations among developmental pathways; Via and Lande 1985) may cause the intermediate growth rate and development time that we observed.

It is interesting to note that although patterns of pupal mass according to latitudinal origin in the common-garden experiment followed a geographical pattern, the highest mass occurred at the lowest latitudes (Figure 4a). The field data show no such size pattern in either the first or the second generation, for these latitudes (compare Figure 3b—c with Figure 4a). Moreover, this pattern of higher mass at lower latitudes is not reflected by longer development times, the basic trade-off assumed by Roff (1980, 1983), but rather the opposite. Instead the more time-stressed butterflies at lower latitude grew faster (Figure 4c), achieving high mass in a short time. This can be seen as an example of counter-gradient variation (Conover and Schultz 1995), but not (as is typical) in relation to field temperatures in the source populations, as this would have lead to the opposite growth rate cline. This is noteworthy because counter-gradient variation in response to temperature and time stress often co-vary and cannot readily be decoupled; this is the situation in, for instance, the common frog *Rana temporaria* where frogs from Northern Sweden grow faster (Laugen et al. 2003). It should be noted that the three sites for the common-garden experiment did not include such a time-stressed high latitude population, where there is just enough time for a single generation. At such sites we would expect laboratory growth rates to increase again, as in Alaskan *Papilio canadensis* butterflies when compared to Michigan populations at the same temperature (Ayres and Scriber 1994).

### Combining the evidence

We suggest that the discrepancy between field and laboratory patterns in *P. icarus* can be ultimately explained by the fact that body size is the complex product of growth rate and development time (Wiklund et al. 1991; Abrams et al. 1996; Nylin and Gotthard 1998; Davidowitz and Nijhout 2004) and proximately by the constant laboratory environment. In the field, seasonal cues are available to signal the progress of the season and sometimes the need to cut development short at the expense of final size. In the common-garden experiment such cues were absent (other than a constant long photoperiod and high temperature) suggesting that such cues of high summer induced direct development in all individuals. Pupal mass was then free to reach some value that was, at least in part, a function of larval growth rates. Shorter photoperiods were not included within the scope of the present study, because the expected variation in diapause induction within and among sites - with concurrent life history variation as noted above — would have added further to the complexity of interpretation. Short photoperiods can be expected to speed up growth in individuals destined for direct development, but not do so to the same extent in those destined for diapause (Wiklund et al. 1991; Nylin 1992; Leimar 1996).

Similarly, the observed interaction between sex and site of origin for pupal mass (not seen in the field material) and growth rate demonstrates how observations of sexual size dimorphism in the laboratory may in part be the result of variation in growth rate, rather than reflecting selection on mass *per se*. This suggests that the lack of sexual differences in growth rate and mass - or even reversed sexual difference - in the rearing of the northern population (Figure 4a and c) reflects release from selection for protandry under direct development in this univoltine population (Wiklund et al. 1991). Here, males can achieve protandry by simply breaking diapause early; hence in obligate univoltine populations there is no need for high larval growth rate in males. It has previously been shown for *P. icarus* (Fiedler and Hölldobler 1992; Leimar 1996) that the degree of sexual size dimorphism in this species is plastic. It has been demonstrated here that such laboratory results may not translate directly into size variation under natural conditions, although undoubtedly they demonstrate the presence of sexual differences in reaction norms for life history traits, and can illuminate how life history decisions are taken according to sex.

Despite evidence of growth rate variation in the laboratory, offsetting the correlation between final size and development time assumed by Roff (1980, 1983), in the field we could still see a saw-tooth pattern fully consistent with his predictions. *P. icarus* is a relatively large insect restricted by season length and, hence, is predicted to follow the converse to Bergmann's rule, and its extension, the saw-tooth theory (Chown and Gaston 1999; Blanckenhorn and Demont 2004). Evidently it does so in the field albeit not in a common environment. The grasshopper *Chorthippus brunneus* provides an interesting comparison. In this species, time-constrained northern populations grow faster but have also evolved a lower critical mass (fewer instars) and thus shorter development time and lower adult mass (Telfer and Hassall 1999), in line with both Roff's mechanistic assumptions and with his theoretical predictions. In another grasshopper, *Omocestus viridulus*, populations from different altitudes grow at the same rate but again insects from time-stressed populations have both a shorter development time, fewer instars and lower final mass in the laboratory (Berner and Blanckenhorn 2006). Future studies may reveal whether this stronger role for genetic variation in development time, as measured in the laboratory, is a general pattern explaining size variation in grasshoppers. An indication that this may perhaps not be the case is the fact that a review of intraspecific variation in the number of larval instars in insects (Esperk et al. 2007) found that such variation only rarely functions to couple development time positively with adult mass, as envisaged by Roff (1983) and as found in these grasshoppers.

## Conclusions

In conclusion, it is clear that the study of latitudinal and altitudinal patterns in size and other life history traits is far from complete. There is little agreement even on what is the most common pattern in a given group of animals, let alone on how the patterns are created by genetic and/or plastic variation. As the investigation described here shows, the developmental processes behind the observed phenotypic patterns, even when they correspond to predictions from basic life history theory, can be more complex than assumed by such theory. Size differences between developmental pathways illustrate the role of phenotypic plasticity in these processes, but simultaneously create problems for researchers already at the stage of establishing what the field and laboratory patterns really look like. To understand local life history adaptation we need both a larger database and more studies where field data is combined with the results of common-garden experiments.
